# Comparative Study on Blast Damage Features of Reinforced Concrete Slabs with Polyurethane Sacrificial Cladding Based on Different Numerical Simulation Methods

**DOI:** 10.3390/polym14183857

**Published:** 2022-09-15

**Authors:** Zhidong Liu, Xiaohua Zhao, Da Liu, Gaohui Wang, Mingsheng Shi

**Affiliations:** 1 Yellow River Laboratory, Zhengzhou University, Zhengzhou 450001, China; 2 Hubei Key Laboratory of Blasting Engineering, Jianghan University, Wuhan 430056, China; 3 Jiangxi Province Institute of Water Sciences, Nanchang 330029, China; 4 State Key Laboratory of Water Resources and Hydropower Engineering Science, Wuhan University, Wuhan 430072, China

**Keywords:** polyurethane sacrificial cladding, reinforced concrete slab, contact explosion, damage features, blast mitigation

## Abstract

The defense effects of sacrificial cladding have been extensively studied in the field of blast resistance. As a polymer material with a cellular structure, polyurethane also has the potential to act as sacrificial cladding due to its good mechanical properties. The purpose of this study is to compare and select a numerical simulation method that is suitable for exploring the blast damage mitigation effect of polyurethane sacrificial cladding on reinforced concrete slabs. To this end, three numerical models were developed using the Fully Coupled Eulerian–Lagrangian (CEL) method, the Arbitrary Lagrangian–Eulerian (ALE) coupling method, and the Smoothed Particle Hydrodynamics and Finite Element Method (SPH–FEM) coupling method, respectively. These three numerical models were used to investigate the damage features of reinforced concrete slabs with polyurethane sacrificial cladding (PU–RCS) under contact explosions. A field test was also carried out to provide a comparison for numerical simulation results. Moreover, the advantages and disadvantages of the three simulation results and the applicability of the three coupled models were discussed. The results show that compared with the CEL model and the ALE coupling model, the SPH–FEM coupling model can better simulate the damage features of PU–RCS, such as the cracks on the bottom surface of the RC slab and the large deformation failure state of polyurethane sacrificial cladding, while the CEL model and the ALE coupling model can simulate the propagation process of shock waves and have a lower computational cost. In conclusion, the SPH–FEM coupling method is the most applicable method for exploring the blast damage features of PU–RCS in this study.

## 1. Introduction

With the continuous occurrence of terrorist attacks, local armed conflicts, and accidental explosions throughout the world, explosive loads have posed a major threat to the safety of engineering structures [1]. Reinforced concrete slabs have been widely used worldwide as the main components of engineering structures. However, blast loads, especially contact blast loads, may cause significant damage to reinforced concrete slabs. The reinforced concrete slabs under contact blast can be severely damaged by burst craters, large deformations, spalling and even penetration. Therefore, to prevent serious damage to reinforced concrete structures, the use of reasonable protection techniques to mitigate the damage effects of contact blast loads on structures is a pivotal issue.

Currently, many scholars are devoted to improving the blast resistance of reinforced concrete structures. For example, many geopolymer composites with good mechanical properties have been developed [2]. Recently, the protective properties and protection mechanisms of sacrificial cladding have attracted extensive research interest from scholars. Sacrificial claddings are usually positioned on the outer surface of the structures to be protected, in order to mitigate the blast loads by reducing their amplitude and increasing the applied time span, while absorbing most of the blast induced energy [3]. Wu et al. [4] investigated the blast damage mitigation performance of aluminum foam cladding on reinforced concrete slabs. Bohara et al. [5] investigated the protection effect of reinforced concrete slabs by re-entrantable auxetic honeycomb sandwich panels under non-contact blast. Rebelo et al. [6] analyzed the energy absorption capacity of 3D printed PLA honeycomb structures when used as sacrificial cladding. Zhao et al. [7] used foamed cement-based materials as sacrificial cladding for tunnel linings and evaluated their protective effects through numerical simulations. A large number of research results mentioned above show that in the choice of sacrificial cladding, people are more inclined to use porous structures [8,9] because of their light weight and good energy absorption effect.

**Figure 1:**
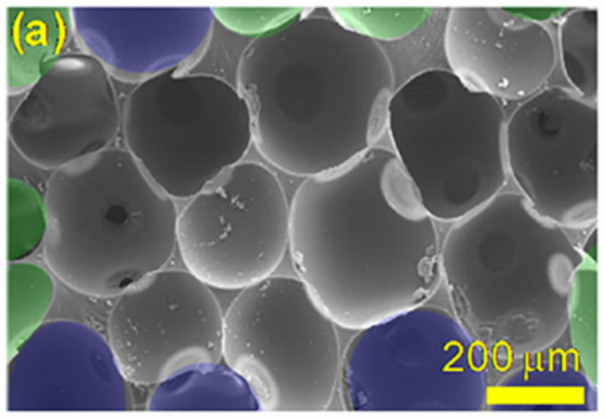
The micromorphology of a PU sample. Reprinted with permission from [18]. Copyright 2020, Elsevier.

Non-water reactive foamed polyurethane (PU), as a polymer material with excellent mechanical properties and cellular structure, also has the potential to act as a sacrificial cladding and play a role in buffering and energy dissipation [10,11]. Figure 1 is a scanning electron microscope image of a PU sample. It can be seen that, at the microscopic scale, the PU sample is made of cell-like vesicle pores and a pure solid polyurethane matrix. The cell-like vesicle pores are often packed together, and they resemble circular shapes with an equivalent diameter of about 80–120 μm. Some research work has been carried out by scholars on the dynamic mechanical properties of polyurethane materials in the fields of impact and explosion. Wang et al. [12,13,14] designed energy-absorbing connectors filled with polyurethane foam and conducted drop hammer impact tests and quasi-static compression tests on them. Combined with finite element simulation, the failure mechanism and energy-absorbing properties were evaluated. Meng et al. [15] investigated the low-velocity impact response of a new steel-polyurethane foam-steel-concrete-steel (SPUFSCS) panel and analyzed the effect of the steel-polyurethane foam layer on the impact resistance of composite panels. Jamil et al. [16] investigated the failure mode of thermoplastic polyurethane sandwich panels under blast loading. Ousji et al. [17] explored the energy absorption capacity and efficiency of low-density polyurethane as sacrificial cladding under blast loading. However, the above series of research results focused on the energy absorption characteristics and failure modes of composite structures containing polyurethane materials. It should be noted that the investigation of the blast damage mitigation effect of polyurethane sacrificial cladding on reinforced concrete structures still needs further research.

There are two main ways to study the damage effects of structures under blast loads, namely field tests and numerical simulation. Field tests can provide the most intuitive and realistic results. However, unlike many static mechanical tests in the engineering field, conducting explosion tests not only requires more professional equipment and strong financial support, but also may endanger the safety of life [19]. With the rapid development of computer technology, numerical simulation has become an important supplement to field tests. However, to reproduce the nonlinear dynamic response of the target structure to the explosion load accurately, a reliable numerical model is required.

In order to integrate various physical processes into a single model system, many complex phenomena need to be considered, which include large deformations near the explosive and fluid–structure interaction problems [20]. To solve these complex problems, many numerical methods have been developed and applied, such as CEL, ALE, SPH, and SPH–FEM, etc. However, the numerical methods that are applicable to different research subjects are not the same. Wang et al. [21,22,23] proposed a fully coupled numerical model based on the CEL method to predict the damage mode of concrete gravity dams under underwater explosion loads and evaluated the blast resistance performance of concrete gravity dams. Meanwhile, reports on the use of the CEL method to simulate the damage characteristics of reinforced concrete slabs under blast loads [24,25,26,27] are also common. One of the reasons why the CEL method is widely used is that it can effectively simulate the fluid–structure coupling problems and maintain high computational efficiency while satisfying the requirements for computational accuracy. Zhao et al. [28,29] used the ALE method to explore the blast resistance ability of SC, SCS, and CSC under contact explosion. Moreover, the SPH method is chosen in the field of large deformations of structures such as soil explosions [30,31]. Yang et al. [32] proposed a coupled SPH–Lagrangian–Eulerian method to describe the damage process of concrete gravity dams under the combined action of intrusion and explosion. Zhao et al. [19] used CEL, SPH, and SPH–FEM methods to simulate the damage features of reinforced concrete slabs under contact explosions, respectively. The comparative analysis of the three methods concluded that the SPH–FEM method is the most effective method to reproduce the damage features of reinforced concrete slabs under contact explosion. At the same time, the SPH–FEM method has been continuously used to solve the problems of the large deformation of reinforced concrete slabs under blast loads [33,34,35,36]. All these studies show that different numerical methods have their own advantages, disadvantages, and applicability conditions, and one of the key factors for the success of numerical simulation is the selection of a suitable numerical method.

The purpose of this study is to compare and select numerical simulation methods that are suitable for investigating the blast damage mitigation effect of polyurethane sacrificial cladding on reinforced concrete slabs. To this end, three fully coupled models were developed using CEL, ALE, and SPH–FEM methods, respectively, to simulate the dynamic response of PU–RCS with an explosive charge of 50 g. A field test was also carried out to verify the accuracy of these three numerical models by comparing the results of the three simulations with the test results. Finally, the advantages, disadvantages, and applicability of each of the three coupled models were compared and analyzed in terms of the damage results and process of PU–RCS, the failure state of the polyurethane sacrificial cladding, the propagation characteristics of the shock wave, and the computational efficiency and cost. The research results are helpful in providing a suitable numerical simulation method for further investigation of the blast damage mitigation performance of polyurethane sacrificial cladding.

## 2. Description of The Coupling Method

With the rapid development of computer technology, numerical simulation has become a powerful means to describe the dynamic response of structures under blast loads. In this paper, three coupling methods, including CEL, ALE, and SPH–FEM, are selected to study the damage features of reinforced concrete slabs with polyurethane sacrificial cladding under contact explosions. In this study, ANSYS/AUTODYN was used to solve the numerical model. As one of the most well-known explicit finite element software packages, it has been widely used for explosion and ballistic analysis.

### 2.1. CEL Method

**Figure 2:**
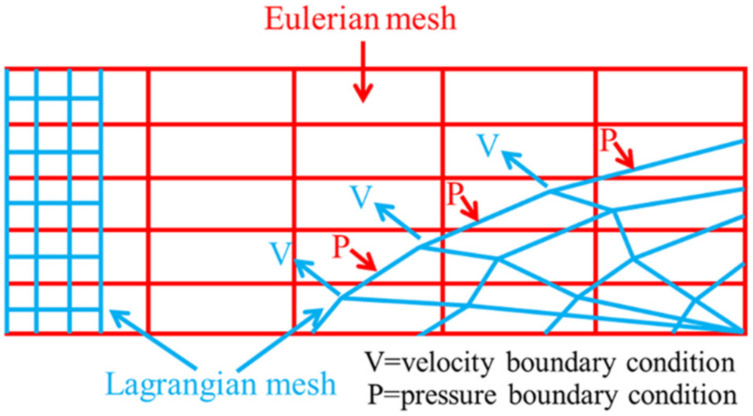
Schematic of the CEL method.

Figure 2 shows a schematic diagram of the CEL method, which is a more commonly used finite element method. In this method, the Eulerian material can impose pressure boundary conditions on the Lagrange mesh, displacing the structure, and the Lagrange interface can pass through the spatially fixed Eulerian mesh in an arbitrary manner. In turn, the Lagrange interface provides velocity boundary conditions for the Eulerian material flow, which cannot penetrate the Lagrange mesh [19,21]. The CEL method is characterized by high computational efficiency.

### 2.2. ALE Coupling Method

**Figure 3:**
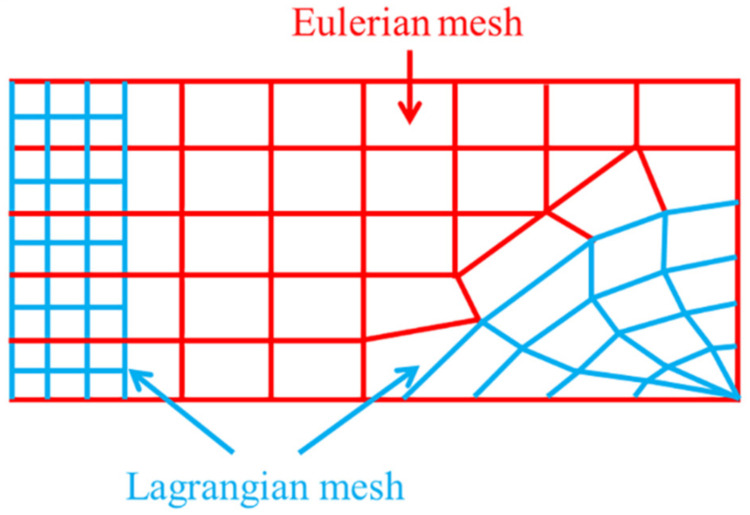
Schematic of the ALE coupling method.

Figure 3 shows the schematic diagram of the ALE coupling method, which also combines the advantages of the Lagrange and Eulerian algorithms [7]. Firstly, it introduces the features of the Lagrange method in the processing of the structural boundary motion so that it can effectively track the motion of the material structure boundary. Secondly, in the division of the internal mesh, it absorbs the advantages of the Eulerian method so that the internal mesh exists independently of the material entity. However, it is not exactly the same as the Eulerian mesh, and the mesh can be properly adjusted in the solution process according to the defined parameters so that the mesh does not have serious distortion. This method is very advantageous when analyzing problems with large deformations of solids, and the material can flow between the meshes when using this method.

### 2.3. SPH–FEM Coupling Method

Smooth particle hydrodynamics (SPH) is a particle-based, meshless form of Lagrangian hydrodynamics, which has been widely used in explosions, penetration, and other problems [30,31]. In the SPH method, the system is represented by a group of particles, which can move freely according to internal particle interaction or external force. An outstanding feature of the SPH method is that it overcomes the calculation termination problem caused by large mesh distortion in mesh-based finite element methods such as the CEL method. Therefore, the SPH method is particularly effective for simulating large deformations caused by explosion loads. However, to obtain accurate simulation results requires a large number of SPH particles, which may lead to lower computational efficiency [19].

**Figure 4:**
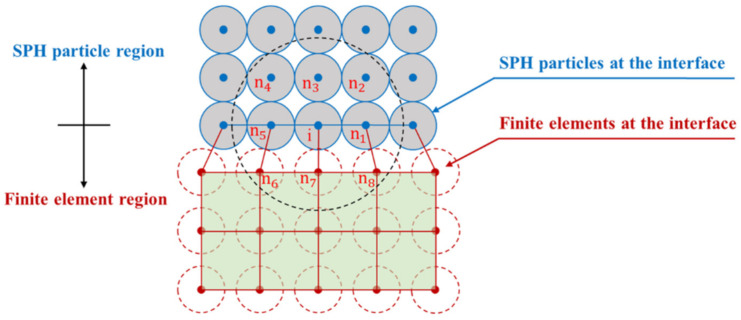
Attachment algorithm for the SPH–FEM coupling method.

To take full advantage of the SPH particles and FEM meshes, people coupled them to simulate the damage and dynamic response of the structure under contact explosion. In the SPH–FEM coupling method, the FEM meshes are used to simulate the structure undergoing small deformations and the SPH particles are used to simulate the structure undergoing large deformations. Figure 4 shows the schematic diagram of the SPH–FEM coupling method. The smaller solid circle, the larger dashed circle, and the smaller dashed circle represent the SPH particle, the support domain of the SPH particle, and the background particle set at the FEM nodes, respectively. The background particles have the properties of SPH particles, while their positions, masses, velocities, and stresses are the same as the corresponding FEM nodes, i.e., the FEM nodes can be considered as SPH particles in the mode of the background particles [19].

**Figure 5:**
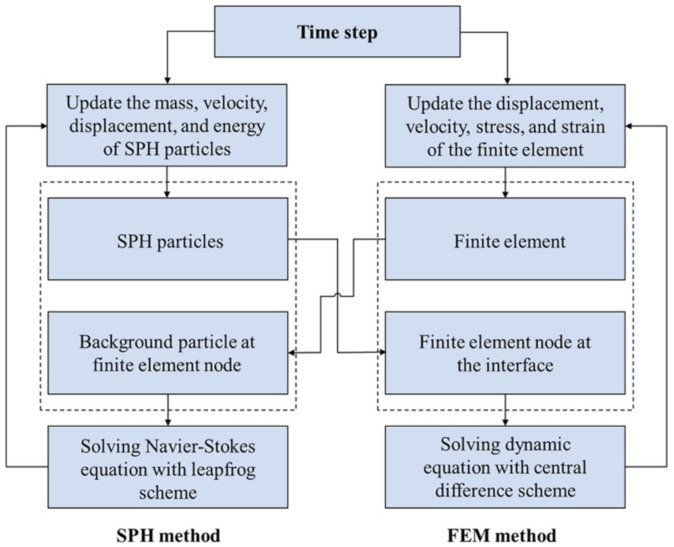
Solution process of SPH–FEM coupling method.

The solution process of the SPH–FEM coupling method is shown in Figure 5. In each time step, the mass, position, velocity, and stress of the finite element node are passed to the corresponding background particle. After ending each time step, the position and velocity of the SPH particle at the interface are passed to the corresponding finite element node. The integration of velocity, displacement, and energy of particle i is calculated considering both particles n_1_, n_2_, n_3_, n_4_, n_5_ and finite element nodes n_6_, n_7_, and n_8_. The background particles are searched by the SPH particles and do not perform SPH numerical integration themselves, and the update of data is performed by the corresponding finite element nodes. The SPH particles provide boundary conditions for the finite elements, while the finite elements enable the SPH particles to avoid boundary effects and maintain the continuity between the SPH particles and the finite elements [34].

## 3. Field Test and Numerical Models

### 3.1. Field Test

**Figure 6:**
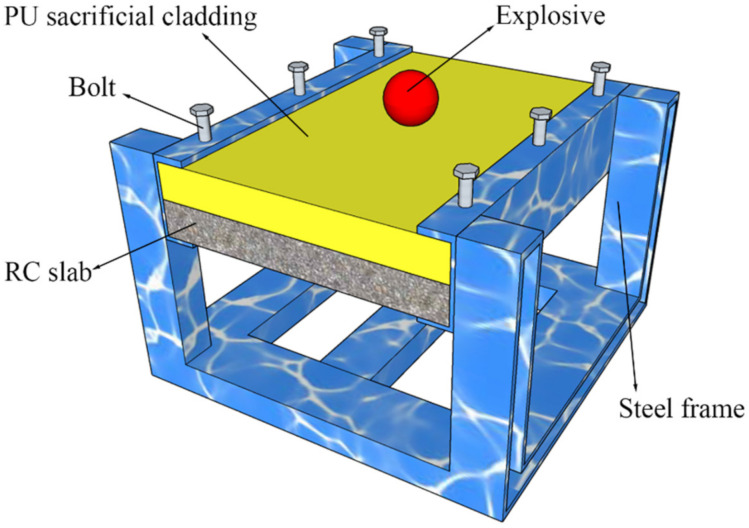
Schematic diagram of the test device.

Figure 6 shows a schematic diagram of the field test device. Before the test, the reinforced concrete slab was installed on the steel frame with both sides of the slab embedded in the grooves, and then the polyurethane plate was placed on the top surface of the reinforced concrete slab to act as sacrificial cladding. By tightening the bolts, the PU–RCS was placed in a boundary condition with both ends fixed. A total of 50 g of explosive was weighed and placed at the center of the top surface of the polyurethane sacrificial cladding. The type of explosive charge used in the test is rock emulsion explosive with a density of 1.05 g/cm^3^, detonation velocity of 4.2–5.0 km/s, and briskness of 12 mm.

**Figure 7:**
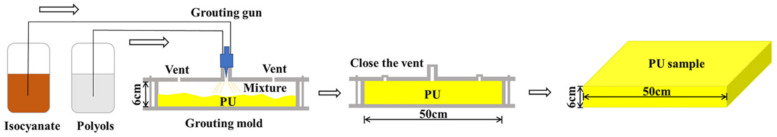
The preparation process of the PU specimens.

Figure 7 shows the preparation process of PU specimens. The PU specimens used are generated by mixing isocyanate and polyol [37] in a weight ratio of 1:1 and then reacting [18]. The density of the prepared specimen is 0.2 g/cm^3^. The design shape is a square plate with dimensions of 50 cm × 50 cm × 6 cm. The Young’s modulus is 35.81 MPa, the elastic limit is 1.54 MPa, and the yield strength is 2.04 MPa. 

**Figure 8:**
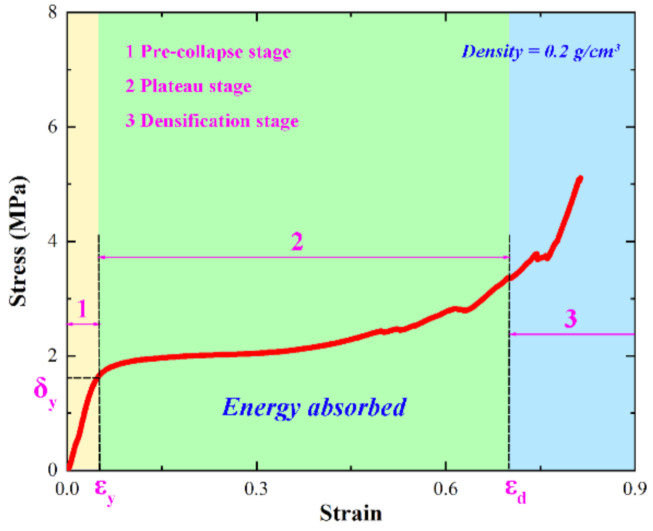
Representative stress–strain curve of PU specimen.

It is well known that the overall energy absorption capacity of the sacrificial cladding is mainly determined by the yield plateau in its stress–strain curve. As shown in Figure 8, the stress–strain curve of non-water reactive foamed polyurethane is mainly divided into three stages, which we refer to as the pre-collapse stage, the plateau stage, and the densification stage, respectively. In Figure 8, $\delta_{y}$ and $\varepsilon_{y}$ are the yield stress and yield strain, respectively, and $\varepsilon_{d}$ is the densification strain. It is not difficult to find that there is a rather long yield plateau between the yield strain $\varepsilon_{y}$ and the densification strain $\varepsilon_{d}$. This indicates that the non-water reactive foamed polyurethane has a strong energy absorption capacity and has the potential to act as sacrificial cladding.

**Figure 9:**
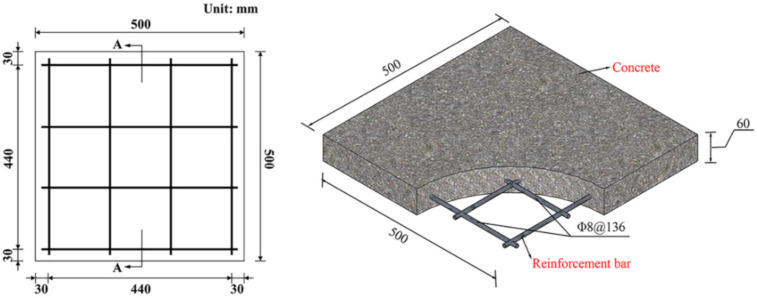
Geometry and reinforcement of the RC slab.

Figure 9 shows the geometric dimensions and reinforcement arrangement of the reinforced concrete slab, and its design size is 500 mm × 500 mm × 60 mm. The strength grade of the selected concrete material is C35, in which the average compressive strength of the concrete is 36 MPa, the tensile strength is 4.0 MPa, and the Young’s modulus is 32 GPa. The strength grade of the reinforcement bar used is HRB400, so the yield strength of the steel bar is 400 MPa. The elastic modulus is 200 GPa. The diameter of the steel bar is 8 mm. The single-layer and two-way reinforcement are arranged, and the thickness of the protective layer of the reinforcement bar is 30 mm.

### 3.2. CEL Model

**Figure 10:**
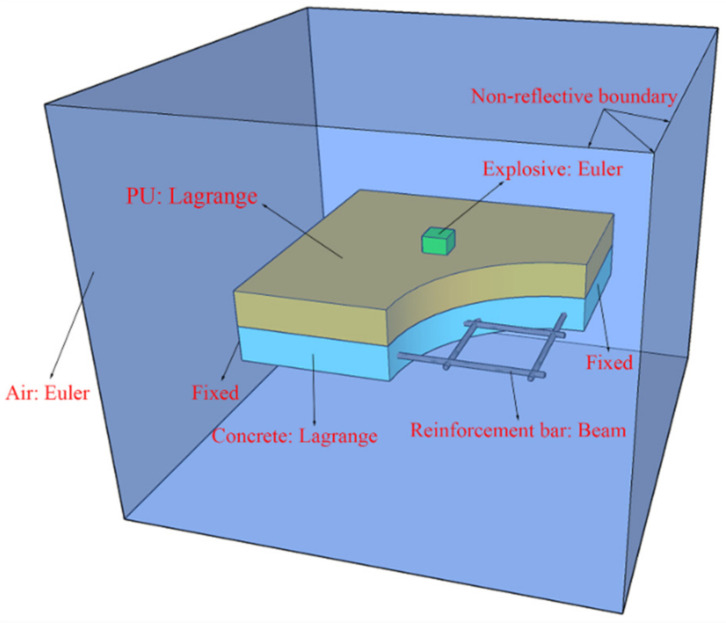
CEL model.

Figure 10 shows the established CEL model. The air is modeled using a Euler subgrid with a mesh size of 10 mm, and the explosive is filled in the Euler meshes. Both the polyurethane sacrificial cladding and the concrete slab are modeled using a Lagrange subgrid with a mesh size of 5 mm. In addition, the reinforcement bars are modeled by beam elements with a mesh size of 5 mm. Shared nodes connections are used between the steel and concrete, and they are assumed to be fully bonded without any slippage. Fixed boundaries are imposed at both ends of the PU–RCS. The boundary condition of the Euler mesh is set as an outflow boundary, i.e., no shock waves are allowed to be reflected at the boundaries of the air domain.

### 3.3. ALE Coupling Model

**Figure 11:**
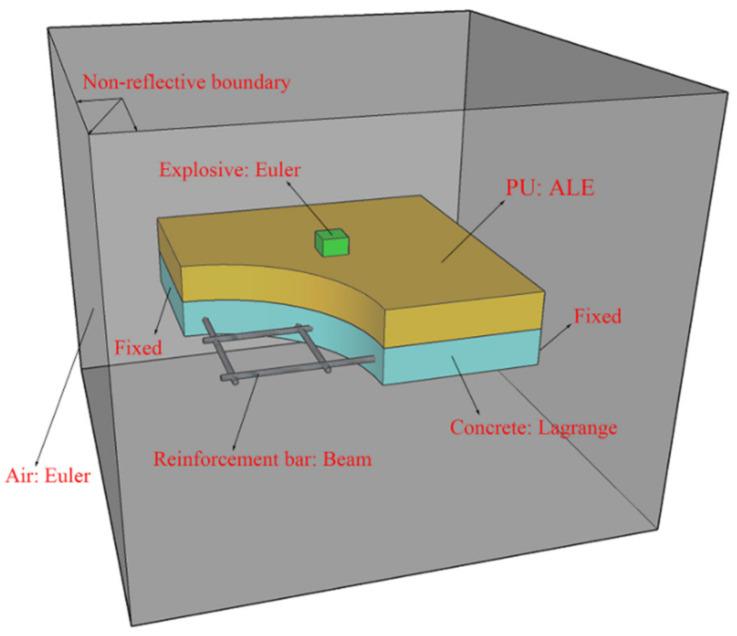
ALE coupling model.

As shown in Figure 11, in the ALE coupling model, the air is also established with the Euler subgrid, and the mesh size is 10 mm. Different from the CEL model, the polyurethane sacrificial cladding is modeled by the ALE subgrid. In addition, the concrete slab is still modeled with the Lagrange subgrid, the reinforcement bars are also modeled with beam elements, and the explosive is filled in Euler meshes. At the same time, the mesh size and quantity, contact type, and boundary conditions of each part are consistent with the CEL model.

### 3.4. SPH–FEM Coupling Model

**Figure 12:**
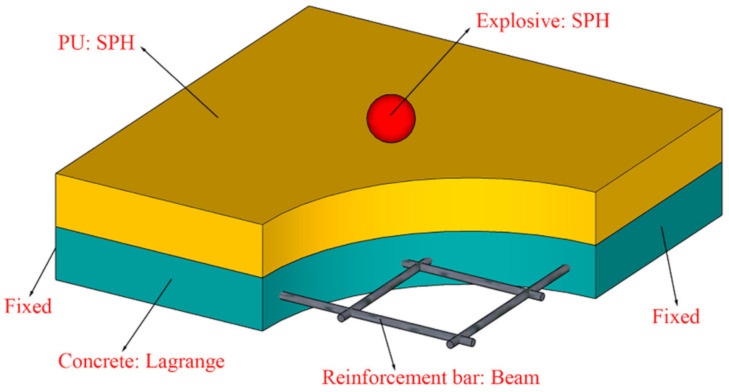
SPH–FEM coupling model.

Figure 12 shows the established SPH–FEM coupling model. In this model, the explosive and the polyurethane sacrificial cladding are modeled by SPH particles. The diameters of the SPH particles constituting the explosive and polyurethane sacrificial cladding are both 5 mm. The concrete is still modeled by the Lagrange subgrid, and the reinforcement bars are modeled by the beam elements, and the size and number of the Lagrange and beam meshes are kept the same as the first two FEM models. It is worth mentioning that air is not considered in the SPH–FEM coupling model. As for the contact explosion, the explosive directly contacts the target structure, so after the explosion, it is mainly the detonation products that damage the target structure. However, the impact of air shock wave on the target structure is relatively small [38]. A fixed boundary is still imposed at both ends of the PU–RCS. Shared node connections are also used between the reinforcement bar and concrete.

The numerical algorithms adopted for each material in the above three coupling models are summarized in Table 1.

### 3.5. Material Model

#### 3.5.1. Concrete

**Figure 13:**
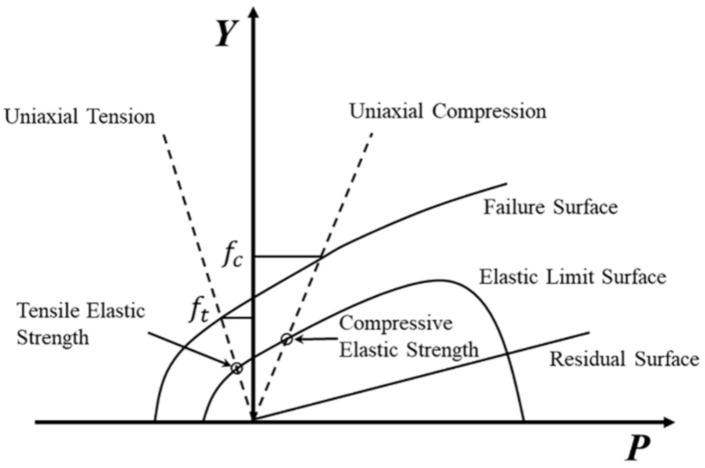
Maximum strength, yield strength, and residual strength surfaces.

In this study, the RHT dynamic damage model was used to describe the behavior of concrete [27]. As shown in Figure 13, the RHT model consists of three pressure-dependent surfaces in the stress space, which are the different limit states of the yield surface: the elastic limit, the failure, and the residual strength, respectively [24].

The failure surface *Y_fail_* is defined as a function of normalized pressure *p*, the lode angle *θ*, and strain rate $\dot{\varepsilon }$.

(1) $$Y_{fail}=Y_{\left(p\right)}\cdot R_{\left(\theta \right)}\cdot F_{RATE\left(\dot{\varepsilon }\right)}$$

(2) $$Y_{\left(p\right)}=f_{c}\left[A\left\{p^{*}-p_{spall}^{*}F_{RATE\left(\dot{\varepsilon }\right)}^{N}\right\}\right]$$

where *f*_c_ is the compressive strength; *A* is the failure surface constant; *N* is the failure surface exponent; $p^{*}$ is the pressure normalized by *fc*; pspall*=ftfc, $f_{t}$ is the uniaxial tensile strength; $F_{RATE\left(\dot{\varepsilon }\right)}$ is the strain rate function; $R_{\left(\theta \right)}$ defines the third invariant dependency of the model as a function of the second and third stress invariants.

The elastic limit surface is strain rate sensitive and scaled from the failure surface; it can be derived as

(3) $$Y_{elastic}=Y_{fail}F_{cap}\left(p\right)F_{elastic}$$

where $F_{elastic}$ is the ratio of the elastic strength to the failure surface strength along a radial path; $F_{cap}\left(p\right)$ is a function that limits the elastic deviatoric stresses under hydrostatic compression.

The residual failure surface can be described as

(4) $$Y_{residual}^{*}=Bp^{*M}$$

where *B* is the residual failure surface constant; *M* is the residual failure surface exponent.

Damage is assumed to accumulate due to inelastic deviatoric straining using the relationships:

(5) $$D=\sum \frac{\Delta \varepsilon_{p}}{\varepsilon_{p}^{failure}}=\sum \frac{\Delta \varepsilon_{p}}{D_{1}{\left(p^{*}-p_{spall}^{*}\right)}^{D_{2}}}$$

where $\varepsilon_{p}$ is the plastic strain; $D_{1}$ and $D_{2}$ are material constants used to describe the effective strain to fracture as a function of pressure. The damage factor *D* between 0 and 1 indicates the concrete element damage ranges from undamaged material to fully damaged material [27]. The material parameters adopted in the present work are shown in Table 2.

#### 3.5.2. Polyurethane

The Crushable Foam model [39] was used to describe the dynamic response behavior of polyurethane material. The stress–strain curve of PU is input to refine its intrinsic parameters. In order to improve the computational efficiency, an instantaneous geometric strain erosion criterion is used, and the deformed unit is automatically removed from the model when the dynamic response of the unit reaches the given criterion. In this study, different erosion criteria for polyurethane materials are simulated in depth, and it is found that satisfactory results are obtained using a geometric strain of 0.3 as the erosion criterion [27].

#### 3.5.3. Steel, Explosives, and Air

The Johnson–Cook model was used to describe the dynamic response behavior of the reinforcement [40]. The model defines the yield stress *Y* as:

(6) $$Y=\left(A+B\varepsilon_{p}^{n}\right)\left(1+C\ln\varepsilon_{p}^{*}\right)\left(1-T_{H}^{m}\right)$$

(7) $$T_{H}=\left(T-T_{room}\right)/\left(T_{melt}-T_{room}\right)$$

where $\varepsilon_{p}$ is effective plastic strain; $\varepsilon_{p}^{*}$ is the normalized effective plastic strain rate; and $T_{H}$ is homologous temperature. *A*, *B*, *C*, *m*, and *n* are material constants. $T_{melt}$ is the melting temperature of the steel; $T_{room}$ is the ambient temperature. The aforesaid material parameters are as follows: *A* = 404 MPa, *B* = 232.4 MPa, *C* = 0.014, *m =* 1.03, *n =* 0.26, $T_{melt}$ = 1793 K, and $T_{room}$ = 300 K, respectively [40].

Explosive charges were modeled using the JWL equation of state [27], and it can be written in the following form:

(8) $$P=A\left(1-\frac{\omega }{R_{1}V}\right)e^{-R_{1}V}+B\left(1-\frac{\omega }{R_{2}V}\right)e^{-R_{2}V}+\frac{\omega E}{V}$$

where *P* is hydrostatic pressure; *V* is the specific volume; *E* is specific internal energy; and *A*, *B*, $R_{1}$, $R_{2}$, ω are material constants. The terms *A* and *B* are the pressure coefficients, $R_{1}$ and $R_{2}$ are the principal and secondary eigenvalues, respectively. ω is the fractional part of the normal Tait equation adiabatic exponent. *e* is a mathematical constant. The aforesaid parameters are *ρ* = 1.05 g/cm^3^, A = 209.7 GPa, *B* = 3.5 GPa, $R_{1}$ = 5.76, $R_{2}$ = 1.29, and *ω* = 0.39, respectively [40].

The ideal gas equation of state [27] was used to model the air. The pressure is related to the energy by

(9) $$P=\left(\gamma -1\right)\rho e$$

where γ is the adiabatic constant for air behaving as an ideal gas; e is the internal energy; $\rho =\rho_{c}/\rho_{0}$, $\rho_{c}$ is the current density, and $\rho_{0}$ is the initial air density. The aforesaid parameters are γ = 1.4, e = 2.068e5 J/kg, and $\rho_{0}$ = 1.225 kg/m^3^, respectively [40].

## 4. Model Validation and Applicability Analysis

### 4.1. Model Validation and Comparison of Damage Features

**Figure 14:**
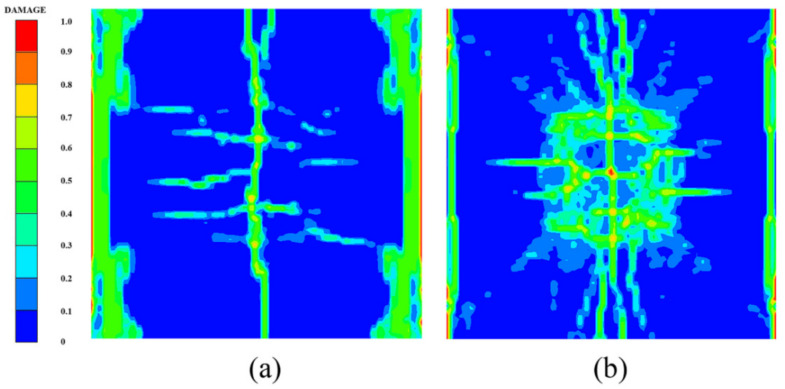
Numerical results of the CEL model: (**a**) top surface; (**b**) bottom surface.

**Figure 15:**
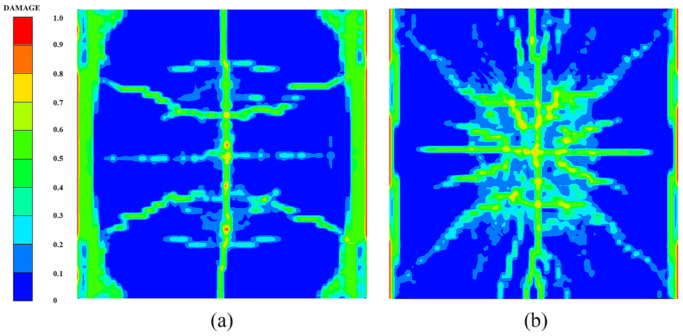
Numerical results of the ALE coupling model: (**a**) top surface; (**b**) bottom surface.

**Figure 16:**
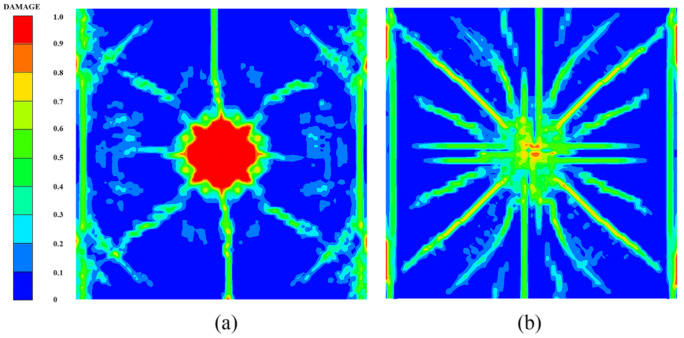
Numerical results of the SPH–FEM coupling model: (**a**) top surface; (**b**) bottom surface.

Figure 14 shows the damage features of the top and bottom surfaces of PU–RCS calculated by the above three coupled models, respectively. It is worth noting that Figure 14, Figure 15 and Figure 16 show the damage results of the reinforced concrete slab after removing the PU sacrificial cladding.

From the damage results of the three models, it can be seen that the top surface of the slab for all three results did not show a deeper burst crater, while a longitudinal crack parallel to the fixed boundary appeared in the middle. The bottom surface of the slab also did not show significant extensive concrete spalling, but rather more diffuse cracks.

Apparently, the simulation results of the CEL model and the ALE coupling model were closer, both of which predicted more severe damage at the fixed boundary on the top surface of the slab. On the other hand, the difference between the CEL model and the ALE coupling model was that the crack distribution predicted by the ALE coupling model was more extensive. Three symmetrical transverse cracks perpendicular to the fixed boundary appeared on the top surface of the slab. The cracks on the bottom surface of the slab showed a dispersive trend from the center to the periphery, while the CEL model did not predict the distribution and trend of such cracks.

Finally, for the SPH–FEM coupling model, it is evident that a circular concrete damage area and four radial cracks appeared in the middle of the top surface of the slab, with a smaller extent of concrete damage at the fixed boundaries on both sides. The most significant difference occurred on the bottom surface of the slab, where the circular concrete spalling area was smaller in the middle, and a large number of divergent radial cracks appeared. These cracks were clearer than those in the first two coupled models.

**Figure 17:**
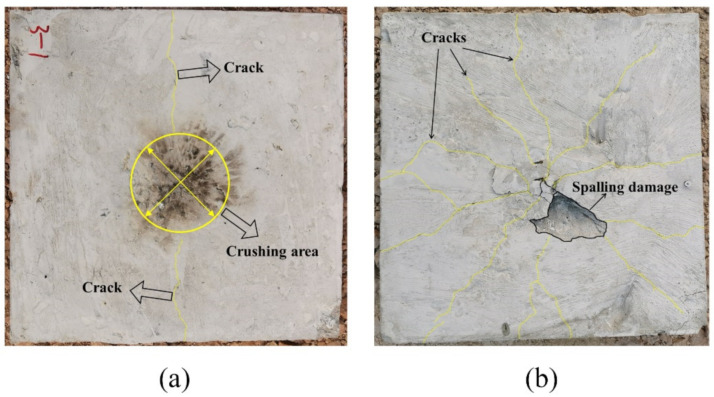
Test results: (**a**) top surface; (**b**) bottom surface.

Figure 17 shows the damage results of the field explosion test. There was a distinct longitudinal crack in the middle of the top surface of the slab and a circular concrete crush area in the center of the slab. A small concrete spalling crater appeared in the middle of the bottom surface of the slab, and the most significant feature was the dispersion of radial cracks almost all over the bottom surface of the slab. It is easy to find that the SPH–FEM coupling model not only predicted the concrete crush zone at the top surface of the slab, but also reproduced the dispersion of cracks from the center to the periphery of the bottom surface of the slab almost perfectly. In addition, the test results showed that the concrete near the fixed boundary on both sides of the slab did not suffer significant damage, which was also consistent with the prediction of the SPH–FEM coupling model.

Compared with the field test results and the prediction results of the SPH–FEM coupling model, both the CEL model and the ALE coupling model underestimated the extent of concrete crushing damage in the middle of the top surface of the slab. However, they overestimated the extent of concrete damage near the fixed boundary of the slab, i.e., both models overestimated the degree of influence of the fixed boundary on the slab damage results. In addition, both models overestimated the extent of spalling damage to the concrete in the middle of the bottom surface of the slab. On the other hand, the CEL model hardly predicted the large number of divergent cracks on the bottom surface of the slab, and the ALE coupling model predicted the tendency of divergent cracks on the bottom surface of the slab, but compared with the SPH–FEM coupling model, the accuracy in terms of the distribution, completeness, and clarity of the cracks was not achieved.

### 4.2. Simulation of the Damage Evolution Process of the RC Slab

**Figure 18:**
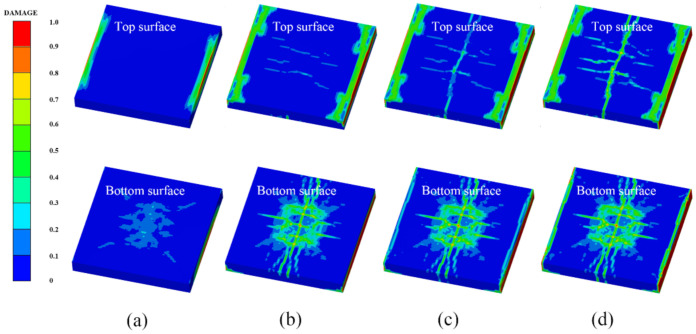
Damage process of the CEL model: (**a**) 0.3 ms, (**b**) 0.9 ms, (**c**) 1.2 ms, (**d**) 2.0 ms.

**Figure 19:**
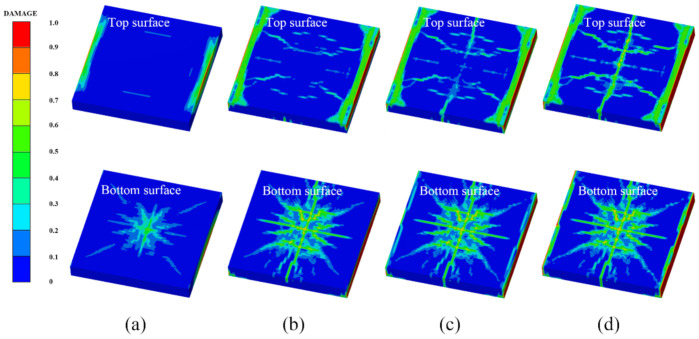
Damage process of the ALE coupling model: (**a**) 0.3 ms, (**b**) 0.9 ms, (**c**) 1.2 ms, (**d**) 2.0 ms.

**Figure 20:**
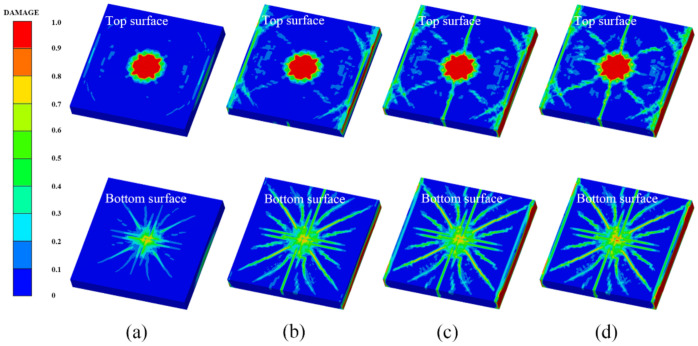
Damage process of the SPH–FEM coupling model: (**a**) 0.3 ms, (**b**) 0.9 ms, (**c**) 1.5 ms, (**d**) 2.0 ms.

Figure 18, Figure 19 and Figure 20 show the damage evolution process of the reinforced concrete slab simulated by the above three coupled models, respectively. It can be seen from the figures that when t = 0.3 ms, the top and bottom surfaces of the slab of the CEL model did not yet show obvious effective damage, while the bottom surfaces of the slab of the ALE coupling model and the SPH–FEM coupling model showed the outline of dispersive cracks. Moreover, the top surface of the slab of the SPH–FEM coupling model showed highly localized crushing damage, but the damage depth was extremely shallow. When t = 0.9 ms, the cracks as well as the spalling damage on the bottom surface of the slab for the three models appeared as more stable contours, while the crack development on the top surface of the slab was incomplete at this time. When t = 1.2 ms, the contours and distribution states of the cracks on the top surface of the slab for the CEL model and the ALE coupling model were relatively clear. However, when t = 1.5 ms, a longitudinal crack finally appeared on the top surface of the slab in the SPH–FEM coupling model.

The damage features of all three coupled models were largely stabilized after t = 2.0 ms, which means that the damage process of PU–RCS by explosion products and blast shock waves had largely ended. In contrast, Zhao et al. [19] and Yang et al. [27] found that the dynamic response of unprotected reinforced concrete slabs under contact explosion was highly localized, with concrete punching and spalling damage expanding rapidly with time. When t = 0.1 ms, serious localized damage had usually occurred on the ordinary reinforced concrete slab, and the damage features had already stabilized after t = 0.6 ms. By comparison, it is easy to find that in this study, due to the good blast mitigation performance of polyurethane sacrificial cladding, the damage effect of reinforced concrete slab was significantly delayed, and the degree of damage was greatly reduced.

### 4.3. Simulation of the Failure State of the Sacrificial Cladding

**Figure 21:**
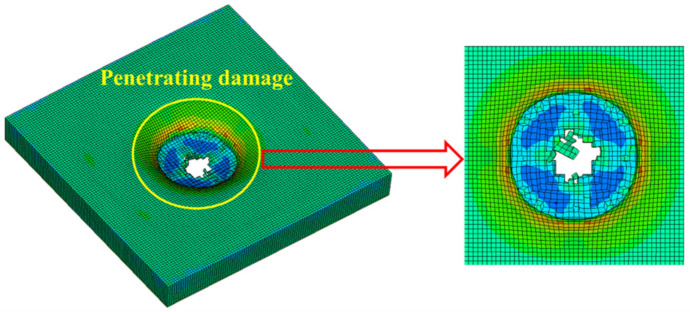
Failure states of polyurethane sacrificial cladding in the CEL model.

**Figure 22:**
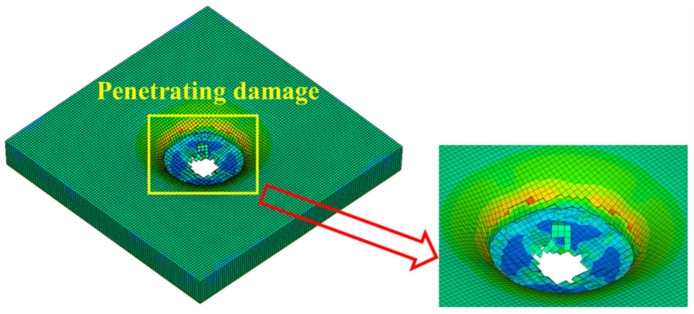
Failure states of polyurethane sacrificial cladding in the ALE coupling model.

**Figure 23:**
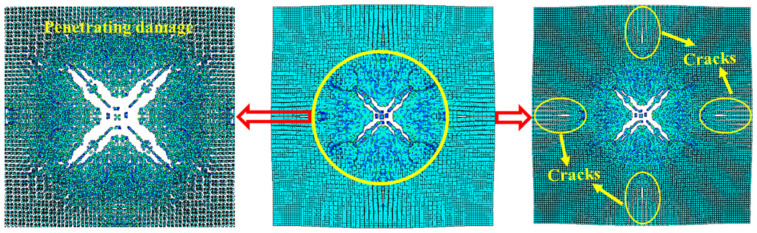
Failure states of polyurethane sacrificial cladding in the SPH–FEM coupling model.

Figure 21, Figure 22 and Figure 23 show the failure states of the polyurethane sacrificial cladding in the above three coupled models, respectively. It can be seen that, although the modeling methods of the polyurethane sacrificial cladding were different in the CEL model and the ALE coupling model, the final failure states of the polyurethane sacrificial claddings in the two models were almost the same, only showing the central penetration damage. However, in the field test, the polyurethane sacrificial cladding also showed extensive fracture damage in addition to the central penetration, which means that the polyurethane sacrificial cladding underwent severe large deformations, and the first two coupled models did not achieve the most ideal results in capturing this large deformation state.

However, compared with the first two coupled models, the polyurethane sacrificial cladding in the SPH–FEM coupling model not only had a greater extent and degree of penetration damage, but also captured the cracks, and its simulation results were closer to the large deformation state of the polyurethane sacrificial cladding in the test. In the SPH–FEM coupling model, the polyurethane sacrificial cladding was modeled with SPH particles, which also validated the view that SPH particles were suitable for simulating structures undergoing large deformation, as mentioned by Wang et al. [20]. In contrast, finite element meshes such as Lagrange and ALE were prone to facing the problems of mesh distortion and entanglement when simulating structures that undergo large deformations, which may lead to the termination of the calculation. Of course, even though the first two coupled models failed to effectively capture the large deformation state of the PU sacrificial cladding, this was not the core problem of this study and did not affect the final damage features of PU–RCS and the damage evolution process.

### 4.4. Simulation of Shock Waves

**Figure 24:**
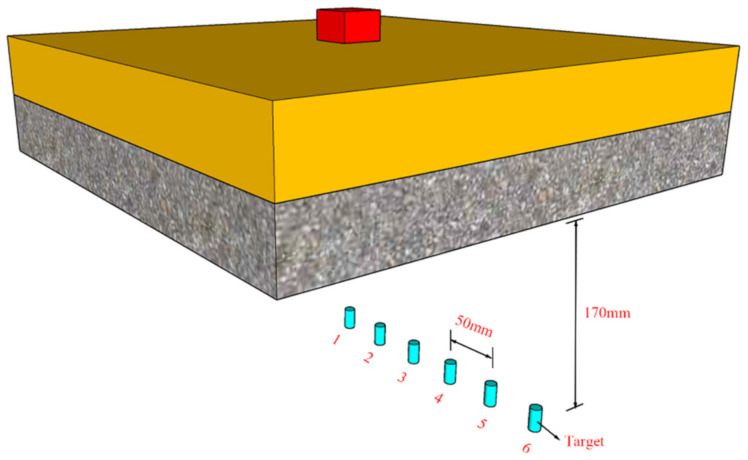
The spatial position of the measuring points.

As shown in Figure 24, in order to simulate the influence of PU–RCS on shock wave propagation under contact explosion, six measuring points were set directly under the PU–RCS in the CEL model and the ALE coupling model, respectively. All measuring points and explosive centers were on the same plane, and this plane was perpendicular to the bottom surface of the PU–RCS. The distance between adjacent measuring points was 50 mm, and the distance between all measuring points and the bottom of the plate was 170 mm. Meanwhile, a free-field explosion without PU–RCS was also set up.

It should be noted that, although the shock wave propagation process was simulated using the CEL model and the ALE coupling model, respectively, only the shock wave pressure curves simulated by the CEL model are given in the paper. This is because the simulation results show that the shock wave pressure curves of both are almost identical. However, since the air is in the default state in the developed SPH–FEM coupling model, this model cannot be used to simulate the propagation process of shock waves at present.

**Figure 25:**
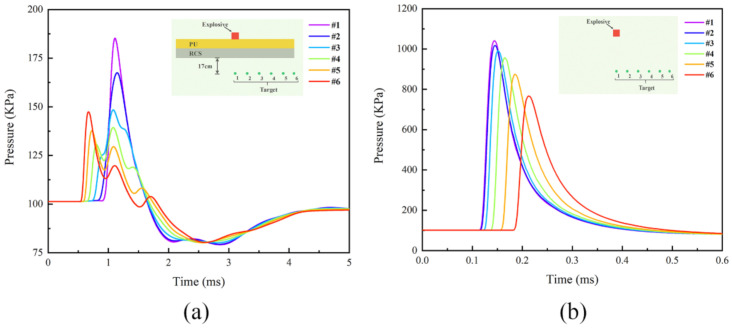
Pressure histories at targets from contact explosion and free field explosion in the CEL model: (**a**) contact explosion; (**b**) free field explosion.

Figure 25 shows the shock wave pressure curves at each target point under contact explosion and free-field explosion, respectively. From Figure 25b, it can be seen that after the detonation of the explosive, the shock wave pressure suddenly rose to its peak and then started to fall back to the atmospheric pressure value approximately exponentially, and the peak pressure of the shock wave decreased with increasing distance from the detonation center. It should be noted that between t = 0.5 ms and 1 ms in Figure 25a was the first time the pressure peak appeared at target points #4, #5, and #6; the second time the pressure peak appeared was shortly after t = 1 ms. The magnitude and time order of the two times of the pressure peaks were exactly the opposite. This phenomenon is named the “bypassing effect”. After the detonation of the explosive, the air above the PU–RCS was strongly compressed by the blast products, resulting in the formation of shock waves. When the shock waves propagated downward, they encountered the obstruction of the PU–RCS. At this time, some of the shock waves would bypass the edge of the slab and continue to propagate downward. Therefore, the target points #6, #5, and #4 near the edge of the slab suffered the brunt of the bypassing effect, resulting in a rapid rise in shock wave pressure, while the target points #1, #2, and #3 near the center of the slab were largely unaffected by the bypassing effect, so their response to pressure peaks was delayed.

**Figure 26:**
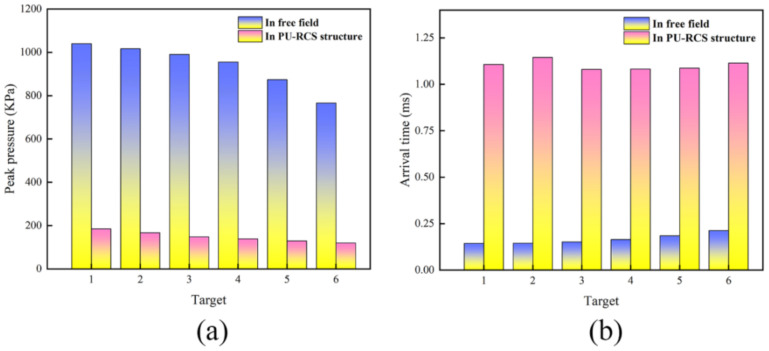
Peak pressure at each target point and its arrival time in CEL model: (**a**) peak pressure at targets; (**b**) arrival time of peak pressure.

In this study, the peak phenomena at target points #4, #5, and #6 due to the bypassing effect can be ignored; instead, focus can be placed on the pressure peaks at the six measuring points after t = 1 ms in Figure 25a. As shown in Figure 26a,b, the pressure peaks at all six measuring points under contact explosion decreased sharply compared to the free field explosion, and there was a significant delay phenomenon in the arrival time of the pressure peaks. This was because a part of the shock wave energy was heavily consumed during the interaction with the PU–RCS, and another part of the shock wave propagated in the opposite direction in the form of the reflected wave. Therefore, the pressure peaks of the shock waves that eventually penetrated the slab and continued to propagate downward were significantly reduced, while the interaction process between the two consumed more time, significantly delaying the moment when the shock waves finally reached the measuring points behind the slab.

### 4.5. Computational Efficiency and Cost

All numerical calculations in this study were performed on a PC with an Intel (R) Core (TM) i7–10700 CPU @ 2.90 GHz processor and 16.0 GB of RAM. In addition, as already mentioned in Chapter 3, the element size corresponding to each part of the structure in the CEL model, the ALE coupling model, and the SPH–FEM coupling model was the same. The end time of the numerical simulation was set to 2.0 ms because the damage characteristics of PU–RCS remained stable after t = 2.0 ms. Under the above-mentioned prerequisites, the computational times of the CEL model, the ALE coupled model, and the SPH–FEM coupled model were about 54,000 s, 57,600 s, and 147,600 s, respectively. The computational time required for the CEL model and the ALE coupling model was approximately the same, while the computational time required for the SPH–FEM coupling model was much longer than the first two models. 

Meanwhile, considering that air was present in the first two coupled models, the overall number of elements was much higher than that of the SPH–FEM coupling model, which further indicated that the computational efficiency of the SPH–FEM coupling model was relatively low. The intrinsic reason for its low computational efficiency is that a large number of SPH particles were used in the establishment of the SPH–FEM coupling model to ensure the computational accuracy. Moreover, the computational efficiency of the SPH particles is lower than that of the finite element mesh with the same number of elements. 

However, compared with the first two models, the modeling process of the SPH–FEM coupling model was relatively simple. Therefore, considering factors such as the simulation accuracy, the complexity of pre-processing and post-processing, and the computational cost, it can be found that the SPH–FEM coupling model is the most suitable for this study.

## 5. Conclusions

The purpose of this study is to compare and select numerical simulation methods that are suitable for investigating the blast damage mitigation effect of polyurethane sacrificial cladding on reinforced concrete slabs. To this end, the dynamic response of PU–RCS under contact explosion was simulated using CEL, ALE, and SPH–FEM methods, respectively. A field test was also conducted to compare the three numerical results with the test results, thus, verifying the rationality of the numerical model. Secondly, the advantages, disadvantages, and applicability of each of the three coupled models were analyzed in terms of the comparison of the damage features and the damage evolution process of PU–RCS; the simulation of the failure state of the polyurethane sacrificial cladding; the simulation of the shock wave; and the computational cost. The following main conclusions can be drawn:

(1) The simulation results of the three coupled models established agree well with the test results, and they can all effectively simulate the damage features of PU–RCS under contact explosion, but the prediction results of the SPH–FEM coupling model are undoubtedly the most accurate, which can achieve a higher degree of accuracy in predicting the distribution range, completeness, and clarity of the cracks.
(2) The three damage evolution processes show that the damage features of PU–RCS basically tend to be stable after t = 2.0 ms, and the damage degree is low, which means that the three models successfully simulated the blast mitigation effect of polyurethane sacrificial cladding. In addition, the SPH–FEM coupling model can accurately reproduce the large deformation failure state of polyurethane sacrificial cladding.
(3) Compared to the SPH–FEM coupling model, both the CEL model and the ALE coupling model can reasonably simulate the influence of PU–RCS on shock wave propagation under contact explosion with a lower computational cost. The existence of PU–RCS severely reduces the peak pressure at the measuring points behind the slab, and the arrival time of the peak pressure is significantly delayed.
(4) The computational cost of the SPH–FEM coupling model is relatively high among the three models. However, the focus of this study is to simulate the damage results of PU–RCS, and the simulation results of the SPH–FEM coupling model have the highest accuracy among the three models. Therefore, the SPH–FEM coupling method is undoubtedly the most suitable numerical method for this study.

## Figures and Tables

**Table 1:** Algorithm of the materials.

Model	Concrete	Polyurethane	Explosive	Air	Rebar
CEL	Lagrange	Lagrange	Euler	Euler	Beam
ALE	Lagrange	ALE	Euler	Euler	Beam
SPH–FEM	Lagrange	SPH	SPH	/	Beam

**Table 2:** Parameters of RHT model for concrete.

Parameter	Value	Parameter	Value
Reference density	$2.75\times 10^{3}\left(\mathrm{kg}/m^{3}\right)$	Tens./Comp. meridian ratio(Q)	0.6805
Porous density	$2.314\times 10^{3}\left(\mathrm{kg}/m^{3}\right)$	Brittle to ductile transition	0.0105
Porous sound speed	$2.920\times 10^{3}\left(m/s\right)$	G (elastic)/(elastic–plastic)	2.000
Initial compaction pressure	$2.330\times 10^{7}\left(\mathrm{Pa}\right)$	Elastic strength/*f_t_*	0.700
Solid compaction pressure	$6.000\times 10^{9}\left(\mathrm{Pa}\right)$	Elastic strength/*f_c_*	0.530
Compaction pressure	3.000	Fractured strength constant *B*	1.600
Bulk modulus *A*_1_	$3.527\times 10^{10}\left(\mathrm{Pa}\right)$	Fractured strength exponent *M*	0.610
Parameter *A*_2_	$3.958\times 10^{10}\left(\mathrm{Pa}\right)$	Compressive strain rate	0.032
Parameter *A*_3_	$9.040\times 10^{9}\left(\mathrm{Pa}\right)$	Max. fracture strength ratio	$1.000\times 10^{20}$
Parameter *B*_0_	1.220	Tensile strain rate	0.036
Parameter *B*_1_	1.220	Erosion strain	Geometric strain
Parameter *T*_1_	$3.527\times 10^{10}\left(\mathrm{Pa}\right)$	Erosion strain	2.000
Parameter *T*_2_	$0\left(\mathrm{Pa}\right)$	Type of geometric strain	Instantaneous
Shear modulus	$1.670\times 10^{10}\left(\mathrm{Pa}\right)$	Damage constant *D*_1_	0.040
Compressive strength (*f_c_*)	$4.000\times 10^{7}\left(\mathrm{Pa}\right)$	Damage constant *D*_2_	1.000
Tensile strength (*f_t_*/*f_c_*)	0.060	Minimum strain to failure	0.010
Shear strength (*f_s_*/*f_c_*)	0.180	Residual shear modulus fraction	0.130
Intact failure surface constant *A*	1.600	Tensile failure	Hydro
Intact failure surface exponent *N*	0.610		

## Data Availability

Not applicable.
